# A Toxicological Evaluation of Mango Leaf Extract (*Mangifera indica*) Containing 60% Mangiferin

**DOI:** 10.1155/2019/4763015

**Published:** 2019-08-01

**Authors:** Robin A. Reddeman, Róbert Glávits, John R. Endres, Amy E. Clewell, Gábor Hirka, Adél Vértesi, Erzsébet Béres, Ilona Pasics Szakonyiné

**Affiliations:** ^1^AIBMR Life Sciences, Inc., 2800 E. Madison St., Suite 202, Seattle, WA 98373, USA; ^2^TOXI-COOP Zrt., Magyar Jakobinusok Tere 4/B, H-1122 Budapest, Hungary

## Abstract

A battery of OECD- and GLP-compliant toxicological studies was performed on mango leaf extract (*Mangifera indica*) containing 60% mangiferin (MLE). No evidence of genotoxicity was found in a bacterial reverse mutation test (Ames). While evidence of clastogenic activity was noted in an in vitro chromosomal aberration test, an in vivo mammalian micronucleus test showed no findings up to the limit dose (2000 mg/kg bw). A 90-day repeated dose oral toxicity study was conducted in rats using doses of 0 (vehicle control), 500, 1000, and 2000 mg/kg bw/day. Based on the lack of mortality or toxic effects in the 90-day study, the NOAEL for MLE in Han:Wist male and female rats was determined to be 2000 mg/kg bw/day, the highest dose tested.

## 1. Introduction

*Mangifera indica* is a large, dome-shaped evergreen tree in the* Anacardiaceae* family that grows to a height of 10-45 meters [1]. It is native to tropical Asia and has been cultivated and all parts of the tree have been used medicinally in India for over 4000 years [1]. The fruits of the mango tree have also been consumed as a food for many hundreds of years in and outside of Asia. Mangiferin (MGF) is a xanthone glycoside found in the leaves, bark, fruit, and roots of* M. indica* and other plants such as* Salacia chinensis, Swertia chirata,* and* Hypericum aucheri* [2–4]. Mangiferin has been characterized as a highly potent antioxidant (polyphenol) and there is some evidence for its activity as an antidiabetic, antimicrobial, antispasmodic, and an antigenotoxic compound [1, 3, 5, 6].

Toxicity studies reporting absence of toxic effects have been performed on a mango stem bark extract containing ~20% mangiferin and 95% mangiferin [7–9] (as described in the discussion section); however, studies on* M. indica* leaf extract containing 60% mangiferin have been lacking. To date, there is one other 90-day study on a MLE containing 62% MGF [10] utilizing dose groups of 100, 300, and 900 mg/kg bw/day; however, it was not reported as adhering to published test guidelines and histopathological examination was not performed. As we are interested in the safety profile of MLE at higher doses, investigation utilizing internationally recognized guidelines, and the determination of a NOAEL for MLE, we submit herein the first in vitro and in vivo toxicological assessment of a mango leaf extract containing 60% MGF (MLE) conducted in accordance with the Organization of Economic Cooperation and Development (OECD) Principles of Good Laboratory Practices (GLP), ENV/MC/CHEM(98)17 [11], and the respective OECD guidelines. As the authors frequently perform this battery of toxicology studies, the methods description partly reproduces wording from other works [12–14].

## 2. Materials and Methods

### 2.1. Chemicals

All chemical reagents, solvents, pharmaceuticals, and other chemicals used in the studies were of analytical or pharmaceutical grade. Agar bacteriological and nutrient broth number 2 were purchased from Oxoid Ltd., (England); biotin, D-glucose-6-phosphate sodium, magnesium chloride, N-2-hydroxyethylpiperazine-N-2-ethane sulphonic acid (HEPES), colchicine, trypsin ethylenediaminetetraacetic acid (EDTA) solution, fetal bovine serum, methyl methanesulfonate (MMS), 2-aminoanthracene (2AA), Dulbecco's modified Eagle's (DME) medium, ethyl methanesulfonate (EMS), cyclophosphamide monohydrate, and antibiotic/antimycotic (penicillin, streptomycin, amphotericin-B) were purchased from Sigma-Aldrich Co., (Germany; USA); monobasic sodium phosphate monohydrate (NaH_2_PO_4_ x H_2_0) was purchased from Carlo Erba, (Val de Reuil, France); potassium chloride (KCl), acetic acid, nutrient agar, Giemsa stain, 4-Nitro-1,2-phenylene-diamine (NPD), dimethyl sulfoxide (DMSO), sodium azide (SAZ), and 9-aminoacridine (9AA) were purchased from Merck KGaA (Germany, Darmstadt); *β*-nicotinamide adenine dinucleotide phosphate (NADP) monosodium salt was purchased from Apollo Scientific (Stockport, UK) and Reanal Private Ltd. (Hungary, Budapest); L-histidine monohydrochloride monohydrate and L-tryptophan were purchased from Acrõs Organics (Belgium, Geel); sodium chloride (NaCl), methanol, and dibasic sodium phosphate dodecahydrate (Na_2_HPO_4_ x 12H_2_O) were purchased from Lachner (Neratovice, Czech Republic); rat liver S9 fraction (sourced from livers of phenobarbital/*β*-naphthoflavone-induced rats) was purchased from Moltox, Inc. (USA); aqua purificata was purchased from Parma Produkt Ltd. (Hungary); Isoflurane CP® was purchased from Medicus Partner, Kft. (Hungary); Cicloplegicedol® eye drops were purchased from Laboratório Edol – Produtos Farmacêuticos S.A. (Portugal); aqua ad iniectabilia was purchased from Naturland Kft, Hungary; and E-Z Mount™ was purchased from Thermo Fisher.

### 2.2. Test Item

The test item was a powdered MLE standardized to contain 60–65% MGF (Zynamite®, (formerly known as Cogniferin™), Nektium; Las Palmas, Spain) as measured by reversed-phase ultra-performance liquid chromatography with photodiode array detection using an external standard for calibration. MLE is made from* M. indica* leaves sourced from trees in China. After harvest, leaves are subjected to grinding, milling, and water/ethanol extraction processes. The extracts are then blended and standardized to contain 60–65% MGF. The remainder of the extract consists of 5% homomangiferin, 15–20% leaf polysaccharides, 5% moisture, and ~5–10% fiber and minerals. The sponsor (Nektium, Spain) provided the test item (lot number MAI6017-0105), and the laboratory verified its identity based on the information provided by the sponsor. Batches of this complex botanical ingredient were prepared just prior to dosing in place of analytical testing.

### 2.3. Test Methods: In Vitro Tests

#### 2.3.1. Bacterial Reverse Mutation Test

The bacterial reverse mutation test was performed to assess the potential mutagenic activity of MLE. The test was conducted in accordance with OECD Test Guideline 471 [15], European Commission Regulation No 440/2008 B13/14 [16], Environmental Protection Agency (EPA) Health Effects Test Guidelines, Office of Prevention, Pesticides, and Toxic Substances (OPPTS) 870.5100, EPA 712-C-98-247 (1998) [17], and International Council on Harmonization Guidance S2(R1) (2012) [18].

The experiments were carried out using histidine-requiring auxotroph strains of* Salmonella typhimurium* TA98, TA100, TA1535, and TA1537 (MOLTOX, Inc., NC, USA) and the tryptophan-requiring auxotroph strain of* Escherichia coli* WP2* uvrA *(MOLTOX, Inc., NC, USA), with and without the metabolic activation system (S9) prepared from the livers of phenobarbital/beta-naphthoflavone-induced rats.

DMSO was chosen as the test item vehicle based on a preliminary solubility test. Based on a preliminary concentration range finding test, the test item was suspended in DMSO to achieve concentrations of 50, 16, 5, 1.6, 0.5, and 0.16 mg/mL for administration of 100 *μ*L per plate resulting in test concentrations of 5000, 1600, 500, 160, 50, and 16 *μ*g/plate. Selection of the concentration range was based on OECD 471 guidelines.

Strain-specific positive controls and solvents were selected according to test guidelines as follows: NPD (4 *μ*g/plate) for TA98, SAZ (2 *μ*g/plate) for TA100 and TA1535, 9AA (50 *μ*g/plate), and MMS (2 *μ*L/plate) in experiments without S9, and 2-AA, 2 *μ*g/plate for* S. typhimurium* strains and 50 *μ*g/plate for* E. coli* WP2* uvrA*, in experiments with S9. Dimethyl sulfoxide served as the vehicle and negative control for 2-AA, 9-AA, and NPD. Ultrapure water served as the vehicle and negative control for MMS and SAZ. The sensitivity, reliability, and promutagen activation potential of the S9 mix utilized in the test were verified by the supplier.

An initial mutation test and a confirmatory mutation test were performed. The initial mutation test consisted of a standard plate incorporation procedure and the confirmatory mutation test consisted of a preincubation procedure. In both procedures cultured bacteria were exposed to the test item and the respective positive and negative controls at all concentrations, with and without S9. The preincubation procedure consisted of short (20 minutes) preincubation of test item and bacteria before plating.

Colony numbers on all test plates were summed by manual counting after which mean values, standard deviations, and mutation rates were calculated. The test item was considered mutagenic if one or both of the following criteria were met:

- A dose-related increase in the number of revertants occurred.
- A reproducible biologically relevant positive response for at least one of the dose groups occurred in at least one strain with or without metabolic activation.

An increase was considered biologically relevant if

- the number of reversions in strains TA98, TA100, or* E. coli* was at least twice as high as the number of reversions of the vehicle control (reversion rate ≥ 2);
- the number of reversions in strains TA1535 or TA1537 was at least three times higher than the number of reversions in the vehicle control (reversion rate ≥ 3).

#### 2.3.2. In Vitro Mammalian Chromosomal Aberration Test

The in vitro chromosomal aberration test was conducted to investigate the clastogenic potential of MLE. The test was performed in accordance with the OECD Test Guideline No 473 [19], and EPA Health Effects Test Guidelines, OPPTS 870.5375 [20].

DMSO served as the solvent for preparing the test item stock solution (100 mg/mL) and as the negative control. Just prior to the test, the stock solution was diluted with DME with and without S9 mix to achieve the test concentrations (listed below). DME was chosen as a solvent due to its compatibility with the test system (V79 male Chinese hamster lung cells, European Collection of Cells Cultures). In a deviation from the test guidelines, the positive control for experiments without S9 was chosen as EMS (1.0 and 0.4 *μ*L/mL) as it is a known mutagen and clastogen and the test facility has a broad historical database. Cyclophosphamide (5 *μ*g/mL) served as the positive control in experiments utilizing S9. Dose selection for the main chromosomal aberration assays was based on a GLP pretest for cytotoxicity.

Subsequently, in Experiment A, cells were exposed to the test item with and without S9 at concentrations of 156.3, 312.5, and 625 *μ*g/mL and to the vehicle control and respective positive controls for three hours and then sampling at 20 hours after treatment. Experiment B was conducted in the same manner as Experiment A except that concentrations of the test item utilized were 19.6, 39.1, and 78.2 *μ*g/mL, the treatment time was 20 hours, and harvesting occurred at 20 and 28 hours after treatment. For the groups treated with S9, cells were exposed to the same concentrations as in Experiment A and the treatment time remained at 3 hours with harvest at 28 hours after treatment. All experiments were conducted in duplicate, utilizing 5 x 10^5^ cells/plate, with concurrent positive and vehicle controls.

All slides were independently coded before blind scoring and analysis of at least 300 well-spread metaphase cells containing 22 ± 2 chromosomes per test item concentration and for negative and positive controls (150 metaphases per slide). Naming and classification of chromosomal aberrations were assigned based upon the International System for Human Cytogenetic Nomenclature [21] and Savage [22, 23]. Toxicity was determined by cell counting from which the relative increase in cell counts was calculated. The CHI^2^ test was utilized for statistical analysis of the data.

After all acceptability criteria were met, a test result was considered to be clearly positive when

- at least one of the test concentrations showed a statistically significant increase in chromosomal aberrations compared to the respective vehicle control;
- the increase was dose related when evaluated with an appropriate trend test;
- any of the results were outside the distribution of the laboratory's historical negative control ranges.

### 2.4. Animal Studies

The Institutional Animal Care and Use Committee of Toxi-Coop Zrt. permitted the conduct of these animal studies which were conducted according to the National Research Council Guide for the Care and Use of Laboratory Animals and in compliance with the principles of the Hungarian Act 2011 CLVIII (modification of Hungarian Act 1998 XXVIII) and Government Decree 40/2013 regulating animal protection.

#### 2.4.1. In Vivo Mouse Micronucleus Test

The in vivo mouse micronucleus test was performed to investigate the potential mutagenic activity of MLE in the bone marrow of male CRL:NMRI BR mice that results in formation of micronuclei in the erythrocytes of treated animals. The test was conducted in accordance with the study plan, OECD Test Guideline 474 [24], and EPA Health Effects Test Guideline OPPTS 870.5395 [25].

Two hours prior to each administration, the test item was formulated in aqua purificata to concentrations of 25 mg/mL, 50 mg/mL, and 100 mg/mL. Cyclophosphamide served as the positive control and was dissolved in aqua ad iniectabilia to a dose concentration of 6.0 mg/mL for intraperitoneal (i.p.) administration of the standard dose volume of 10 mL/kg bw. Aqua purificata and aqua ad iniectabilia served as vehicle controls.

Eight-week-old specific pathogen free Crl:NMRI BR mice, weighing 33.1–35.6 g, were utilized in the study. Acclimatization and husbandry of the animals were carried out in accordance with the cited test guidelines. A GLP preliminary toxicity test was performed to determine the maximum dose level for the main test and to determine if sex differences existed related to toxicity. In the main test, male Crl:NMRI BR mice were randomized into five groups consisting of five animals per group except for the high-dose group into which seven animals were placed for purposes of having two extra animals in case of morbidity.

MLE formulations were administered twice by gavage 24 hours apart at test concentrations of 0 (vehicle control), 500, 1000, and 2000 mg/kg bw at a treatment volume of 20 mL/kg bw. Cyclophosphamide was administered once i.p. at a concentration of 60 mg/kg bw. Animals were observed and examined regularly after dosing until the time of sacrifice for visible signs of reactions to treatment. Twenty-four hours after the conclusion of treatments, and immediately after sacrifice, samples were taken from the bone marrow of both exposed femurs of the animals.

After bone marrow slides were prepared and coded, 4000 polychromatic erythrocytes (PCEs) per animal were scored and evaluated. The proportion of immature to total (immature + mature) erythrocytes was determined by counting a total of at least 500 immature erythrocytes. The frequencies of micronucleated PCEs (MPCEs) in the test and positive control groups were compared to those found in the respective control groups and to laboratory historical control values. Statistical analysis was performed using the Kruskal Wallis Nonparametric analysis of variance test.

#### 2.4.2. 90-Day Repeated-Dose Oral Toxicity Studies in Rats

This 90-day repeated-dose oral toxicity study was conducted to evaluate the possible health hazards, including target organs from repeated exposure to MLE over a 90-/91-day period and to determine a no observed adverse effect level (NOAEL). The study was conducted in compliance with OECD Test Guideline 408 [26] and according to the laboratory's standard operating procedures.

Specific pathogen free Han:Wist rats 46–53 days old and weighing 190–216 g (males) and 135–154 g (females) at the start of the study were randomized according to stratification by body weight into four groups of 10 animals/sex/group (three dose levels and one control group). Animals were acclimatized, housed, and fed per test guidelines.

Dose selection for the 90-day study was based on results from an unpublished 14-day OECD 407 [27] compliant study in which a NOAEL of 2000 mg/kg bw/day, the highest dose tested in male and female Han:Wist rats, was determined. Thus, the test item for the 90-day study was formulated in distilled water (vehicle) to achieve concentrations of 0, 25, 50, and 100 mg/mL for gavage administration of 0 (vehicle control), 500, 1000, and 2000 mg/kg bw/day. All dose formulations were freshly prepared each day and administered within four hours at a constant dosing volume of 20 mL/kg bw.

All clinical observations and examinations were conducted according to the cited guidelines. The functional observation battery (FOB) was conducted based on a modified Irwin test [28]. Individual body weights were recorded and body weight gain was calculated throughout the study. Food consumption was determined, and feed efficiency was calculated at weekly intervals. After an overnight fast following termination of treatment, blood samples were taken from the retroorbital plexus under Isoflurane CP® anesthesia just prior to sacrifice by exsanguination from the abdominal aorta. Clinical pathology examinations consisted of hematology (white blood cell count, neutrophils, lymphocytes, monocytes, eosinophils, basophils, red blood cell count (RBC), hemoglobin, hematocrit, mean corpuscular volume, mean corpuscular hemoglobin (MCH), mean corpuscular hemoglobin concentration (MCHC), and platelets), blood coagulation (prothrombin time (PT) and activated partial thromboplastin time (APTT)), and clinical chemistry (alanine amino transferase, aspartate transaminase, gamma-glutamyl transferase, alkaline phosphatase, total bilirubin, creatine, urea, glucose, cholesterol, inorganic phosphate, calcium, sodium, potassium, chloride, albumin, total protein, and albumin to globulin ratio). Gross pathological examination was performed on every animal on days 90/91 for males/females. External appearance, tissues, and organs were observed macroscopically and selected organs were weighed for each animal and tissues were preserved for potential histopathological examination for the following: adrenal glands, aorta, bone marrow, brain, cecum, colon, duodenum, eyes and optic nerve, epididymides, esophagus, Harderian glands, heart, ileum, jejunum, kidneys, lachrymal glands, liver, lungs, mammary gland, mesenteric lymph nodes, quadriceps muscle, ovaries, pancreas, nasal turbinates, pituitary, prostate, rectum, salivary glands, sciatic nerve, seminal vesicle, skin, spinal cord, spleen, sternum, stomach, submandibular lymph nodes, thymus, thyroid and parathyroid, testes, trachea, urinary bladder, uterus, and vagina. Full histological examination was performed on the preserved organs and tissues of the control and high-dose groups. Histological examination in the low- and mid-dose groups was performed on the basis of macroscopic findings in those groups or in the case of histopathological findings in the high-dose groups.

Statistical analysis was performed with SPSS PC+ software. Heterogeneity of variance between groups was checked by Bartlett's homogeneity of variance test. When no significant heterogeneity was detected, a one-way analysis of variance was conducted and, if positive, a Duncan's multiple range test was used to assess the significance of the difference. When significant heterogeneity was found, the normal distribution of data was evaluated by the Kolmogorov-Smirnov test. When the distribution of data was nonnormal, the nonparametric method of Kruskal-Wallis One-Way analysis of variance was used. If there was a positive result, the intergroup comparisons were performed using the Mann-Whitney U-test. Male and female rats were evaluated separately.

## 3. Results

### 3.1. Bacterial Reverse Mutation Test

The bacterial reverse mutation test met all validity criteria per test guidelines. Positive controls induced the expected revertant colonies over the mean value of the respective vehicle control. No biologically relevant increases were observed in any of the five test strains at any concentration with or without metabolic activation (see Tables 1 and 2).

In the main tests (plate incorporation and preincubation procedures), occasional increases in revertant colony numbers were observed, the highest of which was observed in the initial mutation test in strain TA98 at the highest dose level, 5000 *μ*g/plate, with metabolic activation (mutation rate of 1.88)(see Tables 1 and 2). Contamination occurred on several plates in the preliminary range finding test at the maximum concentration level of 5000 *μ*g/plate with and without S9; therefore, the main experiments were carried out with a total of six parallels instead of in triplicate as the OECD 471 guideline recommends.

### 3.2. In Vitro Mammalian Chromosomal Aberration Test

In Experiment A, one value at the dose of 625 *μ*g/mL without S9 was above the 95% control limits of the historical control data but not statistically significantly different compared to the concurrent vehicle control (see Table 3). In the presence of metabolic activation, results at all test item concentrations were above the 95% control limits of the historical control data; compared to the concurrent vehicle control, the difference was statistically significant and dose related. These changes were considered biologically relevant.

In Experiment B, the frequency of cells having structural chromosomal aberrations without gaps at doses of 78.2 and 39.1 *μ*g/mL without S9 and with treatment/harvest times of 20 h/28 h was above the 95% control limits of the historical control data up to the maximum cytotoxic concentrations (see Table 3). In the experiments with S9, at doses of 312.5 and 625 *μ*g/mL, there were dose-related increases in the number of cells with structural chromosomal aberrations with statistically significantly higher values found at the dose of 625 *μ*g/mL related to the historical control groups and to the concurrent vehicle control. The test met all validity criteria per test guidelines.

### 3.3. In Vivo Mouse Micronucleus Test

The GLP preliminary toxicity test results indicated that no sex differences were present; thus, only male animals were used in the mouse micronucleus test. The highest dose tested of 2000 mg/kg bw did not cause mortality or adverse reactions and was used as the high dose in the main test.

No mortalities and no adverse reactions to treatment were observed in the mouse micronucleus test. Two doses of 500, 1000, or 2000 mg/kg bw of MLE did not induce biologically or statistically significant increases in the frequency of MPCEs in male mice compared to control or historical values (see Table 4). The proportion of immature to total erythrocytes was similar between the 500 and 1000 mg/kg bw dose groups and vehicle controls. The proportion of immature to total erythrocytes was slightly decreased in the 2000 mg/kg bw dose group compared to the vehicle control. The change was not biologically significant but demonstrated exposure of the bone marrow to the test item.

### 3.4. 90-Day Repeated-Dose Oral Toxicity Study in Rats

There were no mortalities in the control, 500, 1000, or 2000 mg/kg bw/day groups during the 90-/91-day (males/females) treatment period. In daily cage-side observations, no clinical signs were observed in control group males, low-dose group males and females, mid-dose group males, or high-dose group females. In the high-dose male group the following clinical signs were observed (observations/10 animals): nuzzling up the bedding material (6/10) from days 31 or 39 transiently (1/10) or up to the end of the treatment period (5/10); slightly decreased activity (2/10) for 10 or 11 days; salivation for three days (1/10); noisy breathing and sneezing (1/10) for 6 and 4 days, respectively; alopecia on the skin of the hind limbs (1/10) between Days 85 and 89. Alopecia was observed on the neck of one control group female on Days 63, 70, 77, 84, and 91 and on the forelimbs of a mid-dose female sporadically between Days 26 and 90. The end of the tail was damaged in one mid-dose female on Days 84 and 91.

Some of the clinical signs of male animals appeared immediately after the administration (nuzzling up the bedding material) or 1–2 minutes thereafter (decreased activity and salivation) and were of short duration (1–2 minutes or 2–3 minutes) after which the animals showed normal behavior and physical condition. Noisy breathing and sneezing were observed transiently in one high-dose male animal. There were no differences between treatment and control groups in the FOB.

Mean body weights and body weight gain in male and female test groups were similar to controls throughout the study period including cumulative body weight gain with the exception of a statistically significantly higher body weight gain in male animals of the low- and mid-dose groups in the last week of the study (Days 84–89) and a statistically significant decrease in body weight gain in high-dose females between Days 21 and 25 (see Supplemental Tables 1 and 2).

With the exception of a statistically significant but slight increase in mean daily food consumption in the low-dose male group between Days 35 and 42, the mean daily food consumption for all test item groups was similar to that of their respective control group throughout the observation period (see Supplemental Table 3). Mean feed efficiency was similar to the respective controls in male and female animals of the 500, 1000, and 2000 mg/kg bw/day groups throughout the treatment period and for the study overall (see Supplemental Table 4). On ophthalmologic examination at the end of the treatment period, no alterations were observed in the high-dose group males and females; thus, animals in the low- and mid-dose groups did not undergo this examination (data not included).

Some statistically significant changes were observed in the high-dose group males on hematological examination (see Table 5 for statistically significant results and Supplemental Table 5 for all hematology results for males and females). Hematological results for female test groups were similar to those for the control group throughout the study. Several statistically significant changes in clinical chemistry were observed in the male and female test groups (see Table 6 for statistically significant results and Supplemental Table 6 for all clinical chemistry results).

Sporadic macroscopic changes were observed in males and females of the control and test item groups with low frequency and/or without dose relationship (see Table 7).

Organ weight evaluations revealed statistically significant changes in absolute weights of the heart, testes, and epididymides in males; however, organ weights relative to brain and body weights were similar to controls (see Table 8 for statistically significant results and Supplemental Tables 7–9 for all organ weight results). Except for a statistically significant increase in the absolute heart weight in low-dose females, absolute and relative organ weights of all female test groups were similar to controls.

Histopathological examination revealed several microscopic lesions in the epididymides, kidneys, livers, skin, or thymus of individual animals (see Table 9). Several lesions were also found upon examination of mid-dose animals with macroscopic lesions. No morphological evidence of degeneration, inflammation, necrosis of the alimentary system, liver, pancreas, cardiovascular system, immune system, hematopoietic system, skeleton, and muscular system, the male and female reproductive system, or the central or peripheral nervous system was observed.

## 4. Discussion and Conclusions

In the current set of studies, a mango leaf extract containing 60% MGF was evaluated for genotoxicity and repeated-dose oral toxicity following OECD guidelines. To the best of our knowledge, this 90-day study is the second 90-day study but the first OECD-compliant one to be performed on a mango leaf extract with similar MGF content (60% and 62%) [10]. Because of the potential for use of MLE in food and supplements for human consumption, this battery of tests was performed and reported herein. Under the conditions of the bacterial reverse mutation test, MLE did not exhibit mutagenic activity. Under the conditions of the performed in vitro chromosomal aberration test, MLE is considered clastogenic. In the in vivo mouse micronucleus test reported herein, MLE did not show any genotoxic activity under the conditions of this test

To our knowledge, the positive in vitro chromosomal aberration test reported herein is the first result showing clastogenic potential for an extract containing MGF. Mango stem bark extract (~20%MGF) has been the subject of various in vitro and in vivo toxicity studies that have reported a lack of genotoxic, clastogenic, acute toxicity, and embryotoxic effects for the extract when tested in a bacterial reverse mutation test (Ames, max dose 5000 *μ*g/plate), in vivo mouse micronucleus test (oral max dose 2000 mg/kg), in vitro human lymphocyte micronucleus test (max dose 1500 *μ*g/mL), acute oral toxicity test (OEDC 423 in rats and mice, 2000 mg/kg), in vivo Comet assay (oral max dose 2000 mg/kg in mice), and an embryotoxicity test (2000 mg/kg in rats) [7–9]. Similarly, MGF isolated from* Salacia chinensis* or mango stem bark extract, having ≥95% purity, has been tested in an Ames test (up to 5000 *μ*g/plate), in vivo (100 mg/kg in mice) and in vitro (10–500 *μ*g/mL) Comet assay, and an in vivo mouse micronucleus assay (2000 mg/kg), all of which showed lack of genotoxic effects [3, 6].

The study on 95% MGF isolated from* Salacia chinensis* included an in vitro chromosomal aberration assay and, while the test was reported as negative for clastogenicity, the test protocols were not clear and data was not shown [3]. Acute toxicity and genotoxicity studies on MGF isolated (≥95% purity) from other plant species have also shown lack of toxic effect for MGF [2, 4]. In addition, MGF has been shown to have protective effects against genotoxicity induced by known mutagenic agents [1, 3, 6].

It is not clear why the current chromosomal aberration test for MLE (60% MGF) showed clastogenicity while the other tests were negative for genotoxic effect. There is evidence that polyphenols, for example, curcumin, can induce chromosomal aberrations under the conditions of the in vitro chromosomal aberration test (mediated by free radical formation from peroxides and via the Fenton system) at lower concentrations but can act as antioxidants under different conditions or at higher concentrations [29–31]. Perhaps this effect is occurring for the polyphenol MGF in MLE as well; further testing would be needed to confirm this assertion.

In the 90-day study, the clinical signs observed in some animals occurred transiently, were of short duration, or low incidence, and/or were likely caused by partial aspiration or misadministration of the test item or mechanical injury. Thus, these signs were not considered toxicologically relevant.

The minor and transient statistically significant changes in body weight gain did not cause statistically significant changes in the mean body weight or cumulative body weight gain of the respective groups of animals. The statistically significant increase in mean daily food consumption in low-dose males was of short duration and did not coincide with any related changes in feed efficiency, body weight, or body weight gain in the animals. Thus, the changes were not considered to be toxicologically relevant.

The statistically significant differences in hematology, blood coagulation parameters, and clinical chemistry were of low magnitude and results remained well within or marginal to the historical control ranges and/or were of low magnitude from controls and without dose relation or related histological findings; thus, the changes were not considered toxicologically significant.

Macroscopic findings in the epididymis, thymus, skin, and spleen occurred in single animals; additionally, per the historical database of the performing laboratory and cited literature, these findings are alterations that occur in untreated experimental rats of this strain at a similar age [32]. Pyelectasia is a species-specific change in experimental rats of this strain [33]; additionally, as there were no signs of inflammation or necrosis this renal change was considered toxicologically not relevant. Hydrometra is a frequent finding in experimental rats [34] and is present in control and test groups at a similar frequency; additionally, there was a lack of related histopathological changes. Thus, these changes were considered not toxicologically relevant.

Statistically significant changes in absolute organ weights were of low magnitude, did not affect relative organ weights, and remained within the historical control ranges; thus, the changes were not considered to be toxicologically relevant.

Changes observed in the epididymides, liver capsules, lungs, skin, spleen, thymus, and uteri occurred in single animals, at the same frequency as in the control group or in the control group only, and/or were considered spontaneous lesions that occur in this age and strain of animal [32, 34, 35]. No test item-related histological findings or target organs were identified; thus, changes were not considered toxicologically relevant.

Statistically significant findings in the 90-day study on a mango leaf extract conducted by Zhang et al. (2014; 100, 300, 900 mg/kg bw/day) [10] for hematology, clinical chemistry, and organ weights have some commonalities with those found in the current study (500, 1000, 2000 mg/kg bw/day) as follows (Zhang et al. (2014) results mentioned first): mean corpuscular hemoglobin was decreased in the 300 mg/kg group males and in the 2000 mg/kg males; total bilirubin was decreased in 900 mg/kg females and in 500, 1000, and 2000 mg/kg males and females; potassium ion was decreased in 300 mg/kg females and 2000 mg/kg females; and epididymides weights were increased in 100, 900 mg/kg males, and 500 mg/kg males. However, there were also statistically significant findings in both studies that did not occur in the other. Overall, Zhang et al. (2014) concluded that the statistically significant findings were not indicative of toxic effects.

Based on the observations made in this 90-day repeated-dose oral toxicity study and the lack of mortality and toxic changes in the examined parameters, the NOAEL for MLE was determined to be 2000 mg/kg bw/day in male and female Han:Wist rats, the highest dose tested.

## Figures and Tables

**Table 1:** Summary of the results of the bacterial reverse mutation test, initial mutation test.

Initial Mutation Test (Plate Incorporation Test)																				
Concentrations (*μ*g/plate)	*Salmonella typhimurium* tester strains																*Escherichia coli*			
	TA 98				TA 100				TA 1535				TA 1537				WP2uvrA			
	-S9		+S9		-S9		+S9		-S9		+S9		-S9		+S9		-S9		+S9	
Mean values of revertants per plate mutation rate (MR)	Mean	MR	Mean	MR	Mean	MR	Mean	MR	Mean	MR	Mean	MR	Mean	MR	Mean	MR	Mean	MR	Mean	MR
Untreated Control	21.0	1.34	17.7	1.06	87.3	1.24	88.0	1.00	15.3	1.00	13.0	1.11	8.7	1.18	8.3	1.09	30.0	1.08	39.3	1.19
DMSO Control	15.7	1.00	16.7	1.00	70.3	1.00	87.7	1.00	15.3	1.00	11.7	1.00	7.3	1.00	7.7	1.00	27.7	1.00	33.0	1.00
*Ultrapure Water Control*	*–*	*–*	*–*	*–*	*68.3*	*1.00*	*–*	*–*	*11.7*	*1.00*	*–*	*–*	*–*	*–*	*–*	*–*	*31.3*	*1.00*	*–*	*–*

5000	22.0	1.40	31.3	1.88	68.2	0.97	86.8	0.99	11.7	0.76	13.3	1.14	7.8	1.07	11.8	1.54	21.3	0.77	19.8	0.60
1600	24.0	1.53	30.3	1.82	68.3	0.97	76.0	0.87	11.3	0.74	11.3	0.97	8.7	1.18	8.3	1.09	25.3	0.92	28.3	0.86
500	20.7	1.32	22.7	1.36	71.3	1.01	69.3	0.79	14.7	0.96	16.3	1.40	6.7	0.91	10.3	1.35	30.3	1.10	32.3	0.98
160	18.0	1.15	19.0	1.14	62.0	0.88	83.3	0.95	11.3	0.74	14.7	1.26	7.7	1.05	8.3	1.09	33.7	1.22	30.7	0.93
50	16.7	1.06	20.3	1.22	75.3	1.07	94.0	1.07	15.3	1.00	12.0	1.03	9.3	1.27	12.0	1.57	28.0	1.01	32.7	0.99
16	20.7	1.32	27.0	1.62	71.3	1.01	102.0	1.16	13.7	0.89	13.7	1.17	10.7	1.45	9.7	1.26	23.7	0.86	36.3	1.10

*NPD (4 μg)16*	*364.0*	*23.23*	*–*	*–*	*–*	*–*	*–*	*–*	*–*	*–*	*–*	*–*	*–*	*–*	*–*	*–*	*–*	*–*	*–*	*–*
*SAZ (2 μg)*	*–*	*–*	*–*	*–*	*797.3*	*11.67*	*–*	*–*	*1162.7*	*99.66*	*–*	*–*	*–*	*–*	*–*	*–*	*–*	*–*	*–*	*–*
*9AA (50 μg)*	*–*	*–*	*–*	*–*	*–*	*–*	*–*	*–*	*–*	*–*	*–*	*–*	*604.7*	*82.45*	*–*	*–*	*–*	*–*	*–*	*–*
*MMS (2 μL) *	*–*	*–*	*–*	*–*	*–*	*–*	*–*	*–*	*–*	*–*	*–*	*–*	*–*	*–*	*–*	*–*	*816.0*	*26.04*	*–*	*–*
*2AA (2 μg)*	*–*	*–*	*1160.0*	*69.60*	*–*	*–*	*1938.7*	*22.11*	*–*	*–*	*152.3*	*13.06*	*–*	*–*	*109.7*	*14.30*	*–*	*–*	*–*	*–*
*2AA (50 μg)*	*–*	*–*	*–*	*–*	*–*	*–*	*–*	*–*	*–*	*–*	*–*	*–*	*–*	*–*	*–*	*–*	*–*	*–*	*154.0*	*4.67*

2AA: 2-aminoanthracene; 9AA: 9-aminoacridine; MMS: methyl methanesulfonate; MR: mutation rate; NPD: 4-nitro-1,2-phenylenediamine; SAZ: sodium azide.

*∗*DMSO was the vehicle of the test item and positive control substances: NPD, 9AA, and 2AA; ultrapure water was the vehicle for the SAZ and MMS. The mutation rates of the test item, untreated, and positive controls were calculated using the data from their respective vehicle controls.

**Table 2:** Summary of the bacterial reverse mutation test, confirmatory mutation test.

Confirmatory Mutation Test (Preincubation Test)																				
Concentrations (*μ*g/plate)	*Salmonella typhimurium* tester strains																*Escherichia coli*			
	TA 98				TA 100				TA 1535				TA 1537				WP2uvrA			
	-S9		+S9		-S9		+S9		-S9		+S9		-S9		+S9		-S9		+S9	
Mean values of revertants per plate mutation rate (MR)	Mean	MR	Mean	MR	Mean	MR	Mean	MR	Mean	MR	Mean	MR	Mean	MR	Mean	MR	Mean	MR	Mean	MR
Untreated Control	22.7	0.86	29.3	0.96	93.3	1.36	99.3	1.24	10.0	0.97	9.7	0.94	7.3	0.92	5.7	1.00	36.7	1.12	35.7	0.85
DMSO Control*∗*	26.3	1.00	30.7	1.00	68.7	1.00	80.3	1.00	10.3	1.00	10.3	1.00	8.0	1.00	5.7	1.00	32.7	1.00	42.0	1.00
*Ultrapure Water Control∗*	*–*	*–*	*–*	*–*	*75.0*	*1.00*	*–*	*–*	*9.7*	*1.00*	*–*	*–*	*–*	*–*	*–*	*–*	*34.3*	*1.00*	*–*	*–*

5000	20.8	0.79	53.0	1.73	71.5	1.04	89.7	1.12	12.7	1.23	9.8	0.95	0.2	0.02	10.5	1.85	31.0	0.95	30.8	0.73
1600	19.3	0.73	36.3	1.18	68.3	1.00	75.7	0.94	11.3	1.10	11.3	1.10	3.7	0.46	4.0	0.71	32.7	1.00	37.0	0.88
500	18.3	0.70	24.0	0.78	79.7	1.16	70.3	0.88	13.7	1.32	10.0	0.97	6.0	0.75	8.3	1.47	39.0	1.19	42.3	1.01
160	17.3	0.66	33.3	1.09	79.3	1.16	76.3	0.95	12.3	1.19	8.7	0.84	9.7	1.21	7.3	1.29	42.3	1.30	34.3	0.82
50	25.3	0.96	37.0	1.21	80.3	1.17	95.3	1.19	15.3	1.48	10.0	0.97	8.0	1.00	7.3	1.29	35.7	1.09	44.3	1.06
16	25.0	0.95	32.3	1.05	85.0	1.24	104.0	1.29	11.3	1.10	12.3	1.19	7.3	0.92	8.3	1.47	31.3	0.96	43.7	1.04

*NPD (4 μg)*	*493.3*	*18.73*	*–*	*–*	*–*	*–*	*–*	*–*	*–*	*–*	*–*	*–*	*–*	*–*	*–*	*–*	*–*	*–*	*–*	*–*
*SAZ (2 μg)*	*–*	*–*	*–*	*–*	*1110.7*	*14.81*	*–*	*–*	*1045.3*	*108.14*	*–*	*–*	*–*	*–*	*–*	*–*	*–*	*–*	*–*	*–*
*9AA (50 μg)*	*–*	*–*	*–*	*–*	*–*	*–*	*–*	*–*	*–*	*–*	*–*	*–*	*372.0*	*46.50*	*–*	*–*	*–*	*–*	*–*	*–*
*MMS (2 μL)*	*–*	*–*	*–*	*–*	*–*	*–*	*–*	*–*	*–*	*–*	*–*	*–*	*–*	*–*	*–*	*–*	*933.0*	*27.17*	*–*	*–*
*2AA (2 μg)*	*–*	*–*	*1434.7*	*46.78*	*–*	*–*	*1120.0*	*13.94*	*–*	*–*	*109.0*	*10.55*	*–*	*–*	*120.3*	*21.24*	*–*	*–*	*–*	*–*
*2AA (50 μg)*	*–*	*–*	*–*	*–*	*–*	*–*	*–*	*–*	*–*	*–*	*–*	*–*	*–*	*–*	*–*	*–*	*–*	*–*	*192.7*	*4.59*

2AA: 2-aminoanthracene; 9AA: 9-aminoacridine; MMS: methyl methanesulfonate; MR: mutation rate; NPD: 4-nitro-1,2-phenylenediamine; SAZ: sodium azide.

*∗*DMSO was the vehicle of the test item and positive control substances: NPD, 9AA, and 2AA; ultrapure water was the vehicle for the SAZ and MMS. The mutation rates of the test item, untreated, and positive controls were calculated using the data from their respective vehicle controls.

**Table 3:** Summary of chromosomal aberration test results.

*Groups*	*Experiment A* ^*1*^						
	S9 Mix	Treatment	Harvest	Mean Aberrant Cells/150 cells		Number of Aberrations	
		Time	Time	incl. gaps	excl. gaps	incl. gaps	excl. gaps
Test Item							
156.3 *μ*g/mL	–	3 h	20 h	9	4	9	4
312.5 *μ*g/mL	–	3 h	20 h	8	4	8	4
625 *μ*g/mL	–	3 h	20 h	13	8	23*∗∗*	14*∗∗*
Vehicle Control	–	3 h	20 h	7	3	7	3
Positive Control	–	3 h	20 h	37*∗∗*	31*∗∗*	56*∗∗*	37*∗∗*
Hist Veh Control^4^	–	3 h	20 h	4.70–7.82	1.59–4.11	n/a	n/a

Test Item							
156.3 *μ*g/mL	+	3 h	20 h	7	5	8	5
312.5 *μ*g/mL	+	3 h	20 h	15*∗*	7	23*∗∗*	12*∗*
625 *μ*g/mL	+	3 h	20 h	18*∗*	10*∗*	24*∗∗*	15*∗∗*
Vehicle Control	+	3 h	20 h	7	3	8	4
Positive Control	+	3 h	20 h	41*∗∗*	36*∗∗*	67*∗∗*	48*∗∗*
Hist Veh Control^4^	+	3 h	20 h	4.66–8.12	1.69–4.35	n/a	n/a

	*Experiment B* ^*2*^						

Test Item							
19.6 *μ*g/mL	–	20 h	20 h	9	3	9	3
39.1 *μ*g/mL	–	20 h	20 h	13	5	13	5
78.2 *μ*g/mL	–	20 h	20 h	14	7	16	7
Vehicle Control	–	20 h	20 h	8	3	8	3
Positive Control	–	20 h	20 h	44*∗∗*	39*∗∗*	67*∗∗*	45*∗∗*
Hist Veh Control^4^	–	20 h	20 h	4.44–7.90	1.60–4.27	n/a	n/a

	*Experiment B* ^*3*^						

Test Item							
19.6 *μ*g/mL	–	20 h	28 h	8	3	8	3
39.1 *μ*g/mL	–	20 h	28 h	12	5	14	6
78.2 *μ*g/mL	–	20 h	28 h	18*∗*	7	19*∗*	7
Vehicle Control	–	20 h	28 h	6	3	7	3
Positive control	–	20 h	28 h	45*∗∗*	35*∗∗*	64*∗∗*	44*∗∗*
Hist Veh Control^4^	–	20 h	28 h	4.31–7.77	1.59–4.11	n/a	n/a

Test Item							
156.3 *μ*g/mL	+	3 h	28 h	11	4	12	5
312.5 *μ*g/mL	+	3 h	28 h	13	7	14	7
625 *μ*g/mL	+	3 h	28 h	21*∗∗*	11*∗*	25*∗∗*	14*∗∗*
Vehicle Control	+	3 h	28 h	7	3	7	3
Positive Control	+	3 h	28 h	43*∗∗*	39*∗∗*	67*∗∗*	45*∗∗*
Hist Veh Control^4^	+	3 h	28 h	4.96–7.56	1.92–4.12	n/a	n/a

^1^Positive controls: (–S9): ethyl methanesulfonate (1.0 *μ*L/mL); (+S9): cyclophosphamide (5.0 *μ*g/mL)

^2^Positive control: (–S9): ethyl methanesulfonate (0.4 *μ*L/mL)

^3^Positive controls: (–S9) ethyl methanesulfonate (0.4 *μ*L/mL); (+S9): cyclophosphamide (5.0 *μ*g/mL)

^4^Numbers reported are the 95% confidence interval

*∗*p < 0.05; *∗∗*p < 0.01, to the concurrent vehicle control and to the historical vehicle control

n/a: not applicable; Veh: vehicle; Hist: historical; incl: including; excl: excluding.

**Table 4:** Summary of mouse micronucleus test results.

Groups		total number of PCE analyzed				
mg/kg bw	Sampling		PCE/ PCE+NCE		MPCE^c^	
n=5	time, hours		mean	± SD	mean	± SD
Vehicle control^a,d^	24	20000	0.53	0.02	5.00	1.22
Test item^d^						
500	24	20000	0.51	0.01	3.80	0.84
1000	24	20000	0.50	0.01	5.00	1.00
2000	24	20000	0.49	0.01	4.80	1.79
Positive control^a^	24	20000	0.36	0.06	128.6^b^	4.56
Historical Vehicle Control	24	4000^c^	n/a	n/a	4.77	0.94

MPCE: micronucleated polychromatic erythrocytes; NCE: normochromatic erythrocytes; PCE: polychromatic erythrocytes

^a^Vehicle control: aqua purificata; positive control: 60 mg/kg bw cyclophosphamide

^b^ p<0.01

^c^MPCE per 4000 PCE

^d^dosing occurred twice in 24 hours.

**Table 5:** Summary of relevant hematological findings for male rats in the 90-day study.

*Group*		*RBC*	*MCH*	*MCHC*	*PT*	*APTT*
mg/kg bw/d		*[x10* ^*12*^ */L]*	*[pg]*	*[g/L]*	*[sec]*	*[sec]*
n=10						
*Males*						
*Control*	Mean	9.55	18.50	356.30	10.28	12.54
	SD	0.11	0.38	3.50	0.26	1.49
*500*	Mean	9.76	18.10	352.90	10.25	12.75
	SD	0.33	0.58	5.34	0.15	1.04
	SS	*∗*				
*1000*	Mean	9.49	18.52	354.40	10.54	13.19
	SD	0.33	0.63	5.25	0.45	1.84
	SS					
*2000*	Mean	9.88	17.83	345.40	10.69	14.70
	SD	0.36	0.34	4.35	0.29	0.97
	SS		*∗∗*	*∗∗*	*∗∗*	*∗∗*
test for significance		U	DN	DN	U	DN
historical control range		7.52-10.21	16.6-18.8	349-379	16.5-27.3	15.2-31.8

APTT: activated partial thromboplastin time; DN: Duncan's multiple range test; MCH: mean corpuscular hemoglobin; MCHC: mean corpuscular hemoglobin concentration; NS: not significant; PLT: platelets; PT: prothrombin time; RBC: red blood cell count; SS: statistical significance; U: Mann-Whitney U-test versus control

*∗* = p < 0.05

*∗∗* = p < 0.01.

**Table 6:** Summary of relevant clinical chemistry findings in the 90-day study.

*Group*		*TBIL*	*GLUC*	*K+*
mg/kg bw/day				
n=10		*[μmol/L]*	*[mmol/L]*	*[mmol/L]*
*Males*				
*Control*	Mean	1.85	5.68	4.61
	SD	0.40	0.34	0.25
*500*	Mean	1.42	5.35	4.51
	SD	0.30	0.35	0.23
	SS	*∗*		
*1000*	Mean	1.46	5.30	4.67
	SD	0.35	0.26	0.17
	SS	*∗*	*∗*	
*2000*	Mean	1.27	5.29	4.62
	SD	0.36	0.47	0.23
	SS	*∗∗*	*∗*	
test for significance		DN	DN	NS
historical control range		0.71-2.79	4.58-8.24	3.84-5.04

*Females*				
*Control*	Mean	2.09	5.33	4.01
	SD	0.40	0.72	0.16
*500*	Mean	1.69	5.07	4.03
	SD	0.47	0.59	0.16
	SS	*∗*		
*1000*	Mean	1.47	5.05	4.02
	SD	0.32	0.40	0.18
	SS	*∗∗*		
*2000*	Mean	1.46	4.89	3.85
	SD	0.33	0.56	0.11
	SS	*∗∗*		*∗*
test for significance		DN	NS	DN
historical control range		1.23-3.30	4.44-7.55	3.42-4.35

DN: Duncan's multiple range test; GLUC: glucose; K+: potassium; n: numbers of animals/group; NS: not significant; SD: standard deviation; SS: statistical significance; TBIL: total bilirubin.

*∗* = p < 0.05

*∗∗* = p < 0.01.

**Table 7:** Summary of macroscopic observations*∗*.

*Organs*	*Dose group mg/kg bw/day*	*Control*	*500*	*1000*	*2000*
	*Observations*	n=10	n=10	n=10	n=10
*Males*					
	No macroscopic findings	10/10	7/10	8/10	9/10
Kidneys	Pyelectasia	0/10	2/10	2/10	1/10
Epididymides	Yellow-green formation	0/10	1/10	0/10	0/10
Diaphragm	*Hernia diaphragmatic*	0/10	0/10	0/10	1/10

*Females*					
	No macroscopic findings	7/10	5/10	3/10	4/10
Thymus	Light orange colored	0/10	1/10	0/10	0/10
Kidneys	Pyelectasia	1/10	0/10	1/10	2/10
Spleen	Adhesion to the intestines	0/10	0/10	0/10	1/10
	Formation	0/10	0/10	0/10	1/10
Uterus	Hydrometra	3/10	5/10	6/10	3/10
Tail	Damage at the end	0/10	0/10	1/10	0/10
Skin	Alopecia	0/10	0/10	1/10	0/10
Diaphragm	*Hernia diaphragmatic*	0/10	0/10	0/10	1/10

*∗*Data represent the number of animals with observations / number of animals examined.

**Table 8:** Summary of relevant findings for absolute organ weights in male and female rats, 90-day study.

*Group*		*Absolute organ weight (g)*					
		*Males*				*Females*	
mg/kg bw/day		*Body*	*Heart*	*Testes*	*Epididymides*	*Body*	*Heart*
n=10		*weight*				*weight*	
* Control*	Mean	442.5	1.10	3.57	1.46	241.6	0.68
	SD	28.94	0.09	0.30	0.13	12.89	0.04
* 500*	Mean	456.2	1.13	3.81	1.64	245.5	0.74
	SD	54.93	0.17	0.21	0.23	18.43	0.05
	SS			*∗*	*∗*		*∗*
* 1000*	Mean	422.3	0.99	3.51	1.46	242.3	0.72
	SD	36.69	0.07	0.25	0.14	15.50	0.05
	SS		*∗∗*				
* 2000*	Mean	442.3	1.07	3.93	1.51	238.5	0.71
	SD	61.46	0.15	0.28	0.15	17.28	0.06
	SS			*∗∗*			
test for significance		NS	U	DN	DN	NS	DN
historical control range		344-488	0.97-1.50	2.58-4.20	1.39-1.93	206-285	0.66-0.96

DN: Duncan's multiple range test; n: number of animals/group; NS: not significant; SD: standard deviation; SS: statistical significance; U: Mann-Whitney U-test versus control.

*∗* = p < 0.05

*∗∗* = p < 0.01.

**Table 9:** Summary of histopathology findings in the 90-day study.

*Organs*	*Observations*	*Incidence of observations per group∗*			
		*Control*	*500*	*1000*	*2000*
			mg/kg bw/day	mg/kg bw/day	mg/kg bw/day
*Males*					
Epididymides	Sperm granuloma (one side)	0/10	1/1	/	0/10
Kidneys	Pyelectasia	0/10	2/2	2/2	1/10
Liver capsule	Focal fibrosis in the Glisson's	0/10	/	/	1/10
Lungs	Alveolar emphysema	1/10	/	/	0/10
	Hyperplasia of BALT	1/10	/	/	1/10
*Females*					
Kidneys	Pyelectasia	1/10	/	1/1	2/10
Liver	Focal fibrosis in Glisson's capsule	0/10	/	/	1/10
Lungs	Alveolar emphysema	1/10	/	/	0/10
	Hyperplasia of BALT	1/10	/	/	1/10
	Bronchitis	0/10	/	/	1/10
Skin	Atrophy of hair follicles (focal)	0/10	/	1/1	0/10
Spleen	Hyperplasia (focal)	0/10	/	/	1/10
Uterus	Dilatation	3/10	/	/	3/10

*∗*incidence: number of animals with observations/number of animals examined

/: not examined; BALT: bronchus associated lymphoid tissue.

## Data Availability

The tabular data used to support the findings of this study are included within this article and in the Supplementary Materials. All experimental records, specimens, and data are archived in compliance with GLP principles in the archives of TOXI-COOP in Balatonfüred, Hungary, at TOXI-COOP in Hungary. Complete summary data tables for the 90-day repeated-dose oral toxicity study are available as Supplementary Materials for male and female rats for the following parameters (reported as means ± standard deviations): body weights, body weight gain, food intake, food efficiency, hematology, clinical chemistry, and absolute and relative (to brain and body weights) organ weights.

## Abbreviations

2AA: 2-Aminoanthracene
9AA: 9-Aminoacridine
BALT: Bronchus associated lymphoid tissue
bw: Body weight
DME: Dulbecco's modified Eagle's
DMSO: Dimethyl sulfoxide
EMS: Ethyl methanesulfonate
EPA: Environmental Protection Agency
FOB: Functional observation battery
GLP: Good Laboratory Practices
i.p.: Intraperitoneal
MGF: Mangiferin
MLE: Mango leaf extract (60% mangiferin)
MPCE: Micronucleated polychromatic erythrocytes
MMS: Methyl-methanesulfonate
NOAEL: No observed adverse effect level
NPD: 4-Nitro-1,2-phenylene-diamine
OECD: Organization of Economic Cooperation and Development
OPPTS: Office of Prevention, Pesticides, and Toxic Substances
PCE: Polychromatic erythrocytes
SAZ: Sodium azide.

## Supplementary Materials

Supplementary Materials Supplementary Table 1: summary of mean body weights in the 90-day study on mango leaf extract (60% mangiferin). Supplementary Table 2: summary of mean body weight gain for the 90-day study on mango leaf extract (60% mangiferin). Supplementary Table 3: summary of mean food consumption for the 90-day study on mango leaf extract (60% mangiferin). Supplementary Table 4: summary of mean feed efficiency for the 90-day study on mango leaf extract (60% mangiferin). Supplementary Table 5: summary of hematology results for the 90-day study on mango leaf extract (60% mangiferin). Supplementary Table 6: summary of clinical chemistry results for the 90-day study on mango leaf extract (60% mangiferin). Supplementary Table 7: summary of mean absolute organ weights for the 90-day study on mango leaf extract (60% mangiferin). Supplementary Table 8: summary of mean organ weights relative to body weight, 90-day study, mango leaf extract (60% mangiferin). Supplementary Table 9: summary of mean organ and body weight relative to brain weight, 90-day study, mango leaf extract (60% mangiferin).
